# Spontaneous regression of advanced transverse colon cancer with remaining lymph node metastasis

**DOI:** 10.1186/s40792-020-00858-1

**Published:** 2020-05-11

**Authors:** Bunpei Nishiura, Kensuke Kumamoto, Shintaro Akamoto, Eisuke Asano, Yasuhisa Ando, Hironobu Suto, Takayoshi Kishino, Minoru Oshima, Masao Fujiwara, Hisashi Usuki, Keiichi Okano, Yasuyuki Suzuki

**Affiliations:** 1 Department of Gastroenterological Surgery, Faculty of Medicine, Kagawa University, 1750-1 Ikenobe, Miki-cho, Kita-gun, Kagawa 761-0793 Japan; 2 Department of Surgery, Sumitomo Besshi Hospital, Niihama, Japan; 3 Department of Surgery, Takamatsu Red Cross Hospital, Takamatsu, Japan

**Keywords:** Spontaneous regression, Advanced colon cancer

## Abstract

**Background:**

The observation of spontaneous regression (SR) has been well documented for many cancer types, including renal cell carcinoma, non-Hodgkin’s lymphoma, leukemia, neuroblastoma, and malignant melanoma. However, the SR frequency in colorectal cancer is very rare. Therefore, the accumulation of SR colorectal cancer cases might contribute to find the regression mechanism.

**Case presentation:**

A 67-year-old woman received colonoscopy due to being positive for fecal occult blood testing and was diagnosed as having a transverse colon cancer at a local hospital. She was admitted to our institution for surgical treatment of the colon cancer. The colonoscopy revealed a type 2 tumor of 13 mm in diameter at the hepatic flexure of the transverse colon. The enhanced computed tomography (CT) showed an enlarged lymph node in the intermediate lymph node region. The 18F-fluorodeoxyglucose positron emission tomography/CT showed no abnormal accumulation on the transverse colon; however, an abnormal accumulation was found at the enlarged lymph node. The patient was preoperatively diagnosed as having advanced transverse colon cancer with lymph node metastasis and underwent laparoscopic right hemicolectomy with D3 lymph node dissection. Pathological examination showed only a scar-like tissue and no cancerous lesion in the transverse colon, while a metastatic lymph node was histologically confirmed in the intermediate lymph node region. Loss of MLH1 and PMS2 expression was observed in the cancer cells of both biopsy specimens and resected lymph nodes. No recurrence was seen for 5 years after surgery.

**Conclusions:**

We reported a rare case of SR of the primary transverse colon cancer without regression of the metastatic regional lymph node. We considered that colorectal cancer with SR should be resected because even if SR of the primary lesion occurs, lymph node metastasis might have an inconsistent behavior as shown in the present case.

## Background

The observation of spontaneous regression (SR) of cancer is mostly reported in renal cell carcinoma, non-Hodgkin’s lymphoma, leukemia, neuroblastoma, and malignant melanoma [1, 2]. Among various cancers, the SR frequency in colorectal cancer is very rare. Although several regression mechanisms of cancer have been speculated [1, 2], it might be difficult to identify accurate mechanism depending on cases. The accumulation of SR colorectal cancer cases might contribute to find the regression mechanism. Recent reports [3–14] showed SR of primary lesions in colorectal cancer without lymph node metastasis. We herein report a rare case of SR of the primary transverse colon cancer without regression of the metastatic regional lymph node.

## Case presentation

A 67-year-old woman received a medical checkup and found a positive fecal occult blood result. Her past medical history was appendicitis and mild pneumonitis. She was referred to a local hospital for further investigation, where colonoscopy revealed a cancer lesion in the transverse colon. Consequently, she was admitted to our institution for surgical treatment.

Physical examination showed no significant findings. Furthermore, the blood test showed no abnormal findings except for mild anemia (Hb 12.9 g/dl). The values of tumor markers were within normal limits (CEA 0.6 ng/ml, CA19-9 31 U/ml).

**Fig.1:**
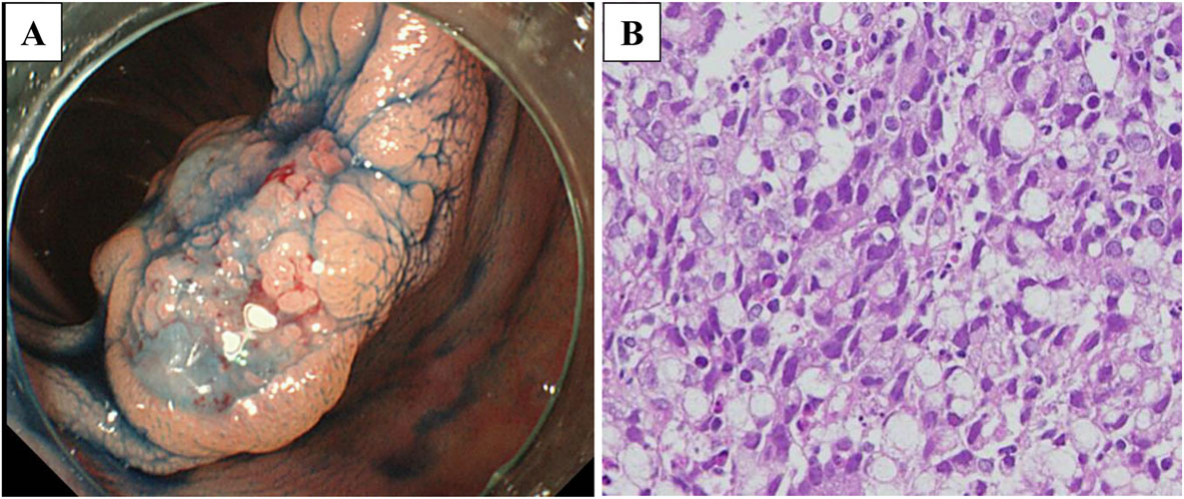
**a** Colonoscopy revealed a type 2 tumor of 13 mm in diameter at the transverse colon. **b** The biopsy specimen showed a poorly differentiated adenocarcinoma

**Fig.2:**
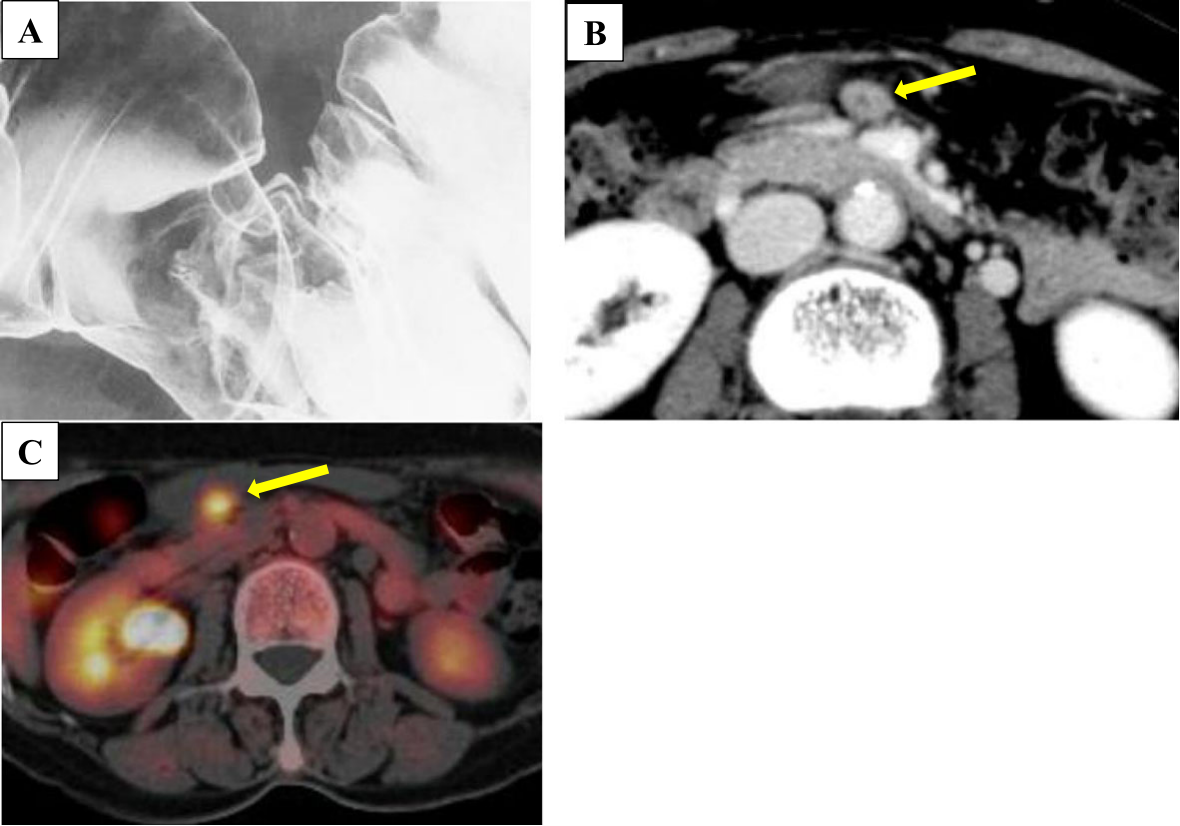
**a** Barium enema examination revealed a trapezoid-formed change at the hepatic flexure of the transverse colon. **b** Abdominal enhanced CT showed an enlarged intermediate lymph node (arrow). **c** PET/CT revealed an abnormal accumulation of SUVmax 3.8 on the intermediate lymph node (arrow).

Colonoscopy was preoperatively performed for the marking neat of the tumor using an ink injection method. The tumor revealed a type 2 lesion of 13 mm in diameter at the hepatic flexure of the transverse colon (Fig. 1a). The histological diagnosis of the biopsy was a poorly differentiated carcinoma with a signet-ring cell carcinoma component (Fig. 1b). Barium enema of the colon revealed a trapezoid-shaped lesion at the same location of the transverse colon (Fig. 2a), leading to the prediction of the wall invasion to MP or deeper. The enhanced computed tomography (CT) could not recognize a tumor in the transverse colon, but an enlarged node was detected in the intermediate lymph node region (Fig. 2b). There was no evidence of distant metastasis.The 18F-fluorodeoxyglucose positron emission tomography (PET)/CT showed an abnormal accumulation of SUVmax 3.8 only on the intermediate lymph node (Fig. 2c). No abnormal accumulation was detected in the transverse colon. Based on these preoperative examination results, she was diagnosed of an advanced transverse colon cancer with lymph node metastasis (T2, N1a, M0, cStageIIIA according to the UICC-TNM classification, 8th edition).

**Fig. 3:**
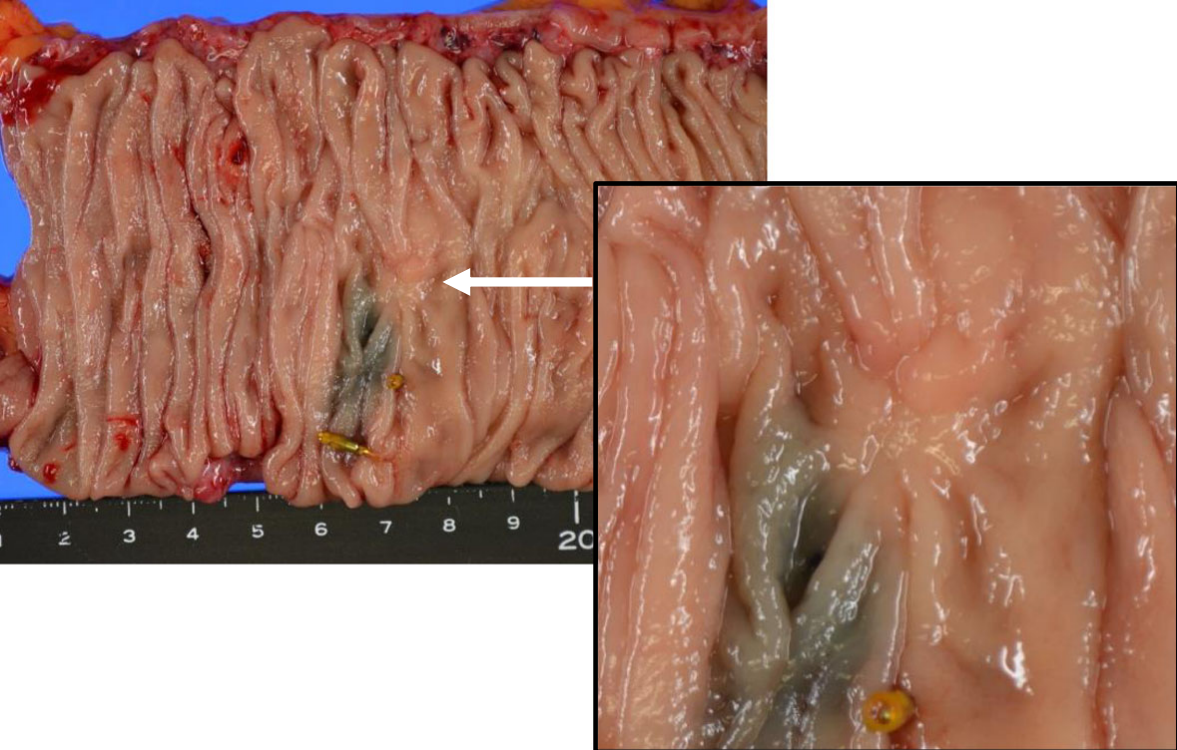
The resected specimen showed only a scar near ink-injected site, but no tumor lesion was detected. Only a scar-like lesion was detected

**Fig.4:**
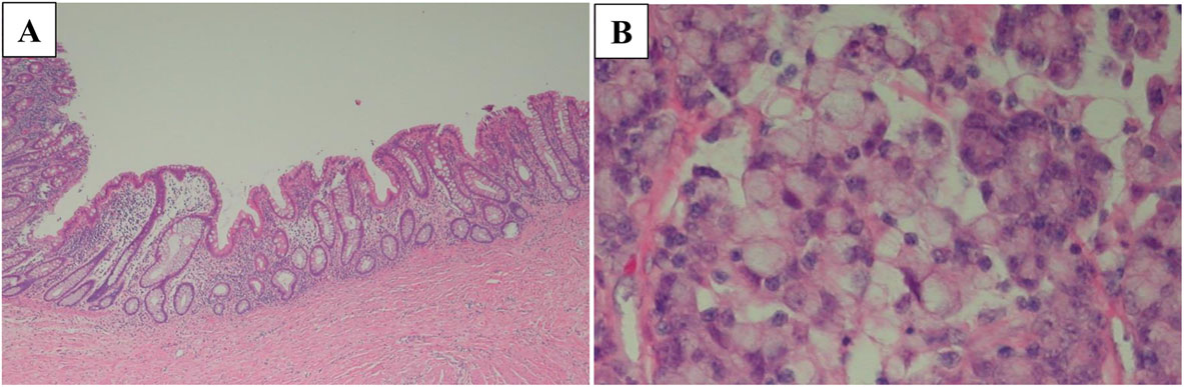
**a** There was inflammation and fibrosis with no cancer cells. **b** Metastatic lymph node was diagnosed as poorly differentiated carcinoma including mucinous component

Laparoscopic right hemicolectomy with D3 lymph node dissection was performed 3 months later after the initial colonoscopy without any treatment, including folk medicine and medication for boost immunity. The enlarged lymph node was located nearby the left branch of the middle colic artery. Macroscopically, the tumor was not observed in the resected specimens (Fig. 3). A scar-like lesion around the preoperative inking was seen in the colonic mucosa (Fig. 3). Pathological findings revealed inflammatory cell infiltration and fibrosis between the submucosa (SM) and the muscularis propria (MP) (Fig. 4a). No malignant cells were identified at the scar lesion, although poorly differentiated carcinoma with mucinous component was pathologically observed in the harvested enlarged lymph node (Fig. 4b).

She was discharged from our institution 9 days after the operation without any complication and was given adjuvant chemotherapy due to regional lymph node metastasis. At 5 years after surgery, there is no evidence of local recurrence and distant metastasis.

## Discussion

To the best of our knowledge, this is the first report of SR case of primary transverse colon cancer in which regional lymph node metastasis was present though there have been several reports of SR primary colorectal cancer without lymph node metastasis. SR has been well documented for many cancer types, including renal cell carcinoma, non-Hodgkin’s lymphoma, leukemia, neuroblastoma, and malignant melanoma [1, 2]. SR of colon cancer is very rare, accounting for less than 2% of such cases [1]. SR of neoplasms is defined as the malignant tumor mass partially or completely disappears without any treatment or as a result of a therapy considered inadequate to influence a systemic neoplastic disease [1, 2]. Abdelrazeq et al. [1] summarized SR cancers, which were reported from the 1900s through 2005, including 11 SR cases of primary colorectal cancer and 10 cases of metastatic lesions. Since almost cases were treated with non-curative operation for colorectal cancer, the residual tumor was expected to increase. However, these patients survived for a long time without any clinical evidence of malignancy. In this period, the diagnostic imaging, including colonoscopy and CT, was not sufficient and the autopsies were not performed to confirm the disappearance of cancer cells.

After the year 2000, 12 cases with SR of a primary colorectal cancer have been reported in Japan (Table 1). In the 13 SR cases including the present case, the median age was 70 years old (range: 60–80 years) and 4 cases were females. The primary sites of colorectal cancer were localized in the cecum (*n* = 1), ascending (*n* = 3), transverse (*n* = 7), and rectum (*n* = 2). Based on these recent reports, the number of right hemi-colon cancer (*n* = 11) was larger than that of left hemi-colon and rectal (*n* = 2) cancer. A previous report [1] showed opposite data that the number of left hemi-colon (*n* = 5) and rectal (*n* = 11) cancer was larger than that of right hemi-colon cancer (*n* = 5). The histological types varied and were not particularly characteristic. The depth of the wall invasion was 6 SM and 7 MP. Lymph node metastasis was not recognized except in the present case. The median duration of the disappearance of the tumor from initial examinations was 2 months, which ranged from 1 to 7 months. These results from the Japanese series after the year 2000 indicated that the SR of colorectal cancer might occur when the tumor is located at the right hemi-colon, the tumor size is up to 30 mm, the wall depth is up to MP, and the operation waiting period is long, regardless of the histological type.

**Table 1:** Reported cases of spontaneous regression of colorectal cancer in Japan

	Author	Reported year	Age	Sex	Primary site	Types	Size (mm)	Histology	Depth	pN	Stage	Duration (month)	Prognosis (month)
1	Kamesui et al. [3]	2000	66	F	Ascending	0-Isp	20	Moderately	SM	0	1	2	12 NR
2	Sakamoto et al. [4]	2009	80	M	Rectum	2	25	Well	MP	0	1	3	24NR
3	Shimizu et al. [5]	2010	80	M	Transverse	2	25	Moderately	MP	-	-	7	64 NR
4	Nakashima et al. [6]	2012	76	F	Caecum	0-Ip	20	Well	SM massive	0	1	2	18 NR
5	Sekiguchi et al. [7]	2013	69	F	Ascending	0-IIa	20	Moderately	SM massive	0	1	1.5	━
6	Nakamura [8]	2013	60	M	Rectum	0-IIa + IIc	10	Well	SM	-	-	1	18NR
7	Serizawa et al. [9]	2015	75	M	Transverse	0-IIc + IIa	15	Well	SM	0	1	2.5	━
8	Kihara et al. [10]	2015	64	M	Transverse	2	30	Moderately	MP	0	1	1.5	12 NR
9	Chida et al. [11]	2017	80	M	Transverse	2	30	Poorly	MP	0	1	1	12 NR
10	Yoshida et al. [12]	2018	73	M	Transverse	0-IIa + IIc	10	Moderately	SM massive	0	1	3	9NR
11	Karakuchi et al. [13]	2019	70	M	Transverse	2	30	Poorly	MP	0	1	2	11 NR
12	Kawakita et la [14].	2019	62	M	Ascending	2	10	Poorly	MP	0	1	1	6 NR
13	Our case	2019	67	F	Transverse	2	13	Poorly	MP	1a	3	3	60 NR

**Fig. 5:**
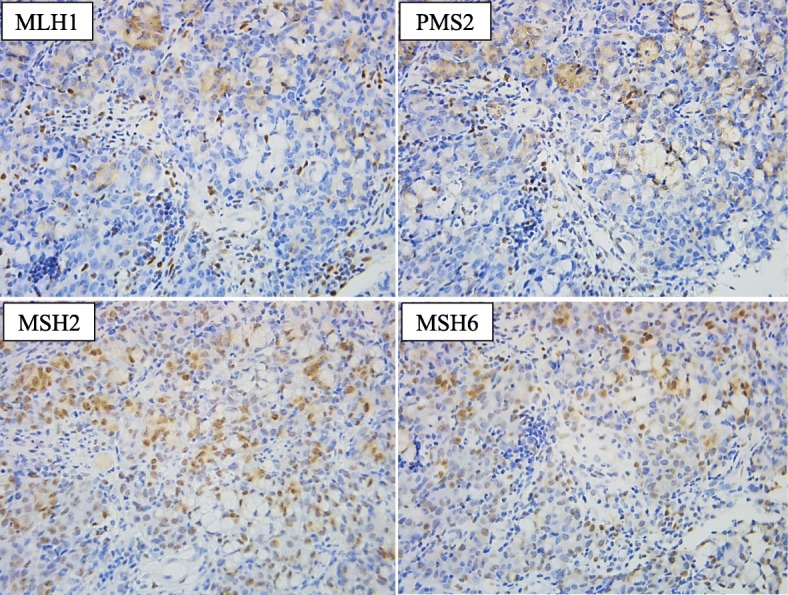
Immunohistochemical staining of indicated mismatch repair proteins in the biopsy specimen from the primary cancer

Although previous reports [1, 2] suggested that the causal factors of SR might include tumor destruction or necrosis by immunological mechanisms or physical stimulation, the exact trigger of SR has not been fully elucidated depending on the cases. Since the frequency of SR has been reported in renal cell carcinoma and malignant melanoma, which are considered to have many neoantigens, an antitumor immune response might be the most likely explanation for the mechanism of SR. As the number of right hemi-colon cancer was larger than that of left hemi-colon and rectal cancer in recent reported cases, we come up with the association of microsatellite instability (MSI). Among colorectal cancer patients, those with MSI-high have generally good prognosis when compared with those with microsatellite stable (MSS) [15]. It could be explained that MSI-high cancer produced many cancer-specific antigens, thereby acquiring the ability of immune response for cancer antigens. Therefore, we investigated the contribution of mismatch repair genes expression in the cancer tissue using an immunohistochemical staining method as described previously [16]. As a result, the loss of MLH1 and PMS2 expression was observed in both primary cancer (Fig. 5) and the metastatic lymph node, suggesting that the abnormality of MLH1 could be affected. There were no past histories of colon cancer or endometrial cancer, and no family history of colorectal cancer, which were suspected in Lynch syndrome. Therefore, MLH1 might lose its function due to acquired predisposition. In our case, the primary lesion might disappear due to the immune response through cancer-specific antigens produced by the loss of MLH1 functions.

However, the primary lesion had SR while the regional metastatic lymph node neither disappeared nor reduced in size. Although observation time might have been insufficient in the present case, it is unlikely that only an antitumor immune response can explain this inconsistency in behavior between the primary and metastatic lesions. It has been reported that the gene expression profiles in metastatic cancer were different from those in primary cancer [17]. Therefore, the immune response could be different between the primary and metastatic cancer, though the MLH1 loss was detected in both the primary cancer and the metastatic lymph node.

It has been reported that SM invasive tumors might disappear by some kind of physical stimulation such as excessive movement due to mechanical cleansing for examinations or the effect of medical examinations, including colonoscopy with biopsy and barium enema [18]. A recent report [18] suggested that the vasoconstriction effect by the epinephrine injection under the SM tumor for endoscopic submucosal dissection could induce SR due to tumor necrosis. Meanwhile, MP invasive colon cancers such as the present case can disappear by physical stimulation remains unclear.

## Conclusion

SR of colorectal cancer is very rare. Therefore, the occasion and treatment strategies have not been established. We considered that colorectal cancer with SR should be resected because even if SR of the primary lesion occurs, lymph node metastasis might have an inconsistent behavior as shown in the present case.

## Data Availability

The authors declare that all the data in this article are available within the article.

## Abbreviations

CT: Computed tomography
MP: Muscularis propria
PET: 18F-Fluorodeoxyglucose positron emission tomography
SM: Submucosa
